# Work-related stress, associated comorbidities and stress causes in French community pharmacies: a nationwide cross-sectional study

**DOI:** 10.7717/peerj.3973

**Published:** 2017-10-26

**Authors:** David Balayssac, Bruno Pereira, Julie Virot, Céline Lambert, Aurore Collin, David Alapini, Jean-Marc Gagnaire, Nicolas Authier, Damien Cuny, Brigitte Vennat

**Affiliations:** 1 Inserm U1107, NEURO-DOL, UFR de Pharmacie, Laboratoire de Toxicologie, CHU Clermont-Ferrand, Délégation à la recherche clinique et à l’innovation, Université Clermont Auvergne, Clermont-Ferrand, France; 2 Délégation à la Recherche Clinique et à l’Innovation, CHU Clermont-Ferrand, Clermont-Ferrand, France; 3 Faculté de Pharmacie, Université Clermont Auvergne, Clermont-Ferrand, France; 4 Inserm U1107, NEURO-DOL, UFR de Pharmacie, Laboratoire de Toxicologie, Université Clermont Auvergne, Clermont-Ferrand, France; 5 Ordre des pharmaciens—Conseil régional Nord Pas de Calais, Lille, France; 6 Ordre des pharmaciens—Conseil régional Auvergne, Clermont-Ferrand, France; 7 Inserm U1107, NEURO-DOL, UFR de Médecine, Laboratoire de Pharmacologie Médicale, CHU Clermont-Ferrand, Université Clermont Auvergne, Clermont-Ferrand, France; 8 CHU Lille, Institut Pasteur de Lille, EA 4483—IMPECS—IMPact de l’Environnement Chimique sur la Santé humaine, Univ. Lille, Lille, France; 9 Faculté de Pharmacie, unité ACCePPT, Université Clermont Auvergne, Clermont-Ferrand, France

**Keywords:** Work-related stress, Community pharmacy, Anxiety, Depression, Fatigue, Sleep disorders

## Abstract

**Background:**

Like other health professionals, community pharmacists are exposed to stress factors (being efficient, avoiding mistakes and bearing emotional load), but they are also under the pressure of entrepreneurial responsibilities. The main objective was to assess the level of work-related stress in French community pharmacies. The other objectives of the study were to assess the associated comorbidities and causes of work-related stress.

**Methods:**

This observational cross-sectional study was sent to all French community pharmacies by email. The survey was anonymous and designed to collect the following items: socio-demographic factors, professional status, characteristics of community pharmacy, work-related stress (visual analogic scale—VAS), fatigue (VAS), sleep disturbances (questions), anxiety and depression symptoms (hospital anxiety and depression scale), medical consultation for work-related stress, medication use for work related stress, psychoactive drug-use and causes of work-related stress. Participants were included in the survey if they were pharmacists (owner or assistant) or pharmacy technicians working in a community pharmacy at the time of the survey. Exclusion criteria were defined as follows: pharmacy students or other professionals involved in a community pharmacy (e.g. dietician, beautician) and lack of professional status information. There was no age limitation.

**Results:**

After three months of data collection, 1,339 participants answered the survey and 1,272 participants were included in conformity with the inclusion and exclusion criteria, and to avoid missing data on the primary endpoint. Work-related stress was detected in 32.8% (417/1,272) of individuals (scores ≥70/100). Men were significantly more affected than women and there was no difference between professional statuses and no relation with the age of the participants. Work-related stress was significantly associated with anxiety, depression, fatigue, sleep disturbances, medical consultations, medication use, alcohol consumption above the WHO recommendations for men and psychoactive drug use. Three causes of stress were clearly identified and related to stress levels, workload, working atmosphere and deterioration of work quality. However, causes of work-related stress were significantly different among professionals, for example: entrepreneurial burden for pharmacists-in-charge and workload for employees (assistant pharmacists and pharmacy technicians).

**Discussion:**

Work-related stress has a very strong impact in French community pharmacies. This stress was associated with several comorbidities and induces health resource consumption. Several causes of work-related stress have been identified such as workload, working atmosphere and deterioration of work quality; however, these causes could be detected and managed to improve stress levels. We recommend developing individual and organizational stress management in French community pharmacies.

## Introduction

According to the definition of the World Health Organization, “*work-related stress is the response people may have when presented with work demands and pressures that are not matched to their knowledge and abilities and which challenge their ability to cope*” (World Health Organization (WHO), 2017). In Europe, 28% of workers (41 million people) suffer from work-related stress (European Union & European Foundation for the Improvement of Living and Working Conditions, 1997). Work-related stress is associated with several illnesses, such as cardiovascular diseases (Fishta & Backé, 2015), musculoskeletal disorders, particularly back pain (Feyer et al., 2000), anxiety, depression (Harvey et al., 2017), fatigue (Kompier, Taris & Van Veldhoven, 2012; Rahman, Abdul-Mumin & Naing, 2016), insomnia (Kompier, Taris & Van Veldhoven, 2012) and alcohol abuse (Colell et al., 2014). It was suggested that more than 10% of occupational diseases are attributed to stress at work (Williamson, 1994). Finally, work-related stress contributes to significant financial losses. For example, the cost of work-related stress was estimated at €20 billion annually in 2002 in Europe (EU-15) while work-related depression was estimated at €617 billion annually in 2013 in Europe (EU-27). These losses result from absenteeism, lost productivity, health care costs and social welfare costs (Hassard et al., 2014; Hassard et al., 2017).

Work-related stress can be induced by several causative factors: those intrinsic to the job (workload, prejudice, hazardous conditions, etc.), role in organization (job description, recognition, etc.), career development (promotion, job insecurity, etc.), relationships at work (relations with co-workers or subordinates, responsibilities, etc.) and organizational structure and climate (control on the job, financial difficulties, etc.) (Michie, 2002; Zoni & Lucchini, 2012).

Work-related stress particularly affects health professionals (Boran et al., 2012) and is underestimated and not managed well (Koinis et al., 2015). Health professionals are exposed to specific stress factors such as increasing workload, emotional response to patient suffering, organizational problems and conflicts (Ruotsalainen et al., 2015). The highly regulated working environment of health professionals may also lead to moral distress caused by antagonism between ethical challenges and structural constraints (Astbury, Gallagher & O’Neill, 2015).

Among health professionals, community pharmacists are also exposed to specific stress factors related to pharmacy work, such as being efficient, avoiding mistakes and the emotional load of patients and families. In addition, they are also entrepreneurs burdened by business fragility and administrative responsibilities. Moreover, in Europe, the world of community pharmacies is changing with budget cuts, deregulation and regulatory changes (Piquemal & Destelle, 2014; Pharmaceutical Group of the European Union, 2015; Tsao et al., 2016; Taylor-Kroll & Hope, 2016; Jacobs, Johnson & Hassell, 2017). Work-related stress has been episodically assessed in community pharmacies. Studies have highlighted that community pharmacists are exposed to higher levels of workplace stressors than the general working population and other health professionals (Jacobs et al., 2014; Johnson et al., 2014). In community pharmacies, work-related stress has a negative impact on safety and quality related event learning, defined as medication errors that reach the patient and those that are intercepted by pharmacy staff before dispensing (Boyle et al., 2016). Among the causes of work-related stress, work overload seems to be an important stressor in community pharmacies (Johnson et al., 2014), but not specific to pharmacy practice. Specific causes of stress have been identified such as staff (lack of competence, loss of confidence), interruptions (disruption of work flows), lack of breaks (impossibility of taking breaks away from work), pharmacy environment (lack of privacy for both pharmacists and patients), isolation (lack of contact with other pharmacists), patient/public (very demanding and impatient) and difficulties in finding time to complete continuing professional development (McCann, Adair & Hughes, 2009). However, no detailed assessment of work-related stress has been performed on a large cohort of pharmacists. Moreover, the consequences of work-related stress for pharmacists’ health are not known and only few studies have focused on the causes of work-related stress (Jacobs et al., 2014; Astbury, Gallagher & O’Neill, 2015; Tsao et al., 2016; Jacobs, Johnson & Hassell, 2017).

The main objective of this study was to assess work-related stress in French community pharmacies. The secondary objectives were to assess the comorbidities (anxiety, depression, fatigue, sleep disturbances and psychoactive drug use) and causes of work-related stress.

## Methods

### Study design

We conducted a cross-sectional nationwide online survey on community pharmacies to assess work-related stress in pharmacy teams from April 17, 2015 to July 17, 2015 (three months). The address of the online survey was sent by email via the National Order of Pharmacists to all French community pharmacies. A total of 22,104 community pharmacies were counted in France in 2016 (data from the National and the Regional Orders of Pharmacists). Individuals were included in the survey if they were pharmacists (owner or assistant) or pharmacy technicians working in a community pharmacy at the time of the survey. These professionals were defined as the pharmacy team and were asked to answer the online survey. Exclusion criteria were defined as follows: pharmacy students or other professionals involved in a community pharmacy (e.g., dietician, beautician) and lack of professional status information. There was no age limitation.

The study was performed in conformity with the STROBE (Strengthening the Reporting of Observational Studies in Epidemiology) guidelines for reporting observational studies (Von Elm et al., 2007). In compliance with French law, the study was registered with the local correspondent of the French Commission on Information Technology, Data Files and *Civil Liberty* (Commission Nationale de l’Informatique et des libertés, no 0113) and declared to the local ethics committee (Comité de Protection des Personnes sud-est 6, IRB: 00008526). The participants’ consent was obtained with the answer to the survey.

### Survey protocol

The survey was anonymous and designed to record the following items: socio-demographic factors (age, gender, marital status, parentality, tobacco and alcohol consumptions); professional status (pharmacist-in-charge, assistant pharmacist or pharmacy technician, type of employment contract, number of hours worked per week); community pharmacy (number of customers/patients per day in the community pharmacy, number of citizens in the area of the community pharmacy); work-related stress; fatigue; sleep disturbances; anxiety and depression symptoms using the hospital anxiety and depression scale (HADS) (Zigmond & Snaith, 1983); medical consultation for work-related stress, medication use for work related stress (therapeutic class, medical prescription or self-medication), psychoactive drug-use and causes of work-related stress. It was estimated that the online survey would take approximately 20 min to complete.

Work-related stress was assessed by a visual analogic scale (VAS, 0-100) with a cut-off threshold of 70/100, as described by Lesage & Berjot (2011) and Lesage, Berjot & Deschamps (2012). Fatigue was also assessed with a VAS (0–100). No cut-off threshold was found in the literature. Sleep disturbances were assessed by specific questions (sleep difficulties, nocturnal waking, waking too early, lack of quality sleep and feeling of lack of sleep) and frequency of sleep disorders (<1/month, <1/week, 1/week, >1/weeks and each night). Anxiety and depression were assessed through the HADS questionnaire. The HADS questionnaire was designed to assess anxiety and depressive disorders in patients. This questionnaire is a 14-item questionnaire divided into two subscales: seven items for anxiety and seven items for depression. For each subscale, a total score ≤7 is considered normal, 8–10 is borderline or suggestive of possible anxiety/depression, and ≥11 is indicative of mood disorder or pathology (Zigmond & Snaith, 1983).

Participants were asked to check proposed causes of work-related stress. Causes of work-related stress were defined in order to cover each aspect of work in a community pharmacy: patient-related (patients are more demanding; too many patients per day; loss of patients’ confidence), community pharmacy-related (fear of dispensing wrong medication; not recognized by other health professionals; fear of competition; future career uncertainty; deterioration of work quality) and job-related (frequent interruptions; too much paperwork; salary not representative of work; overwhelmed; not supported by colleagues/superiors/family; dissatisfied with working atmosphere).

The study data were collected and managed using REDCap electronic data capture tools hosted at CHU Clermont-Ferrand (Harris et al., 2009). REDCap (Research Electronic Data Capture) is a secure, web-based application designed to support data capture for research studies, providing: (1) an intuitive interface for validated data entry; (2) audit trails for tracking data processing and export procedures; (3) automated export procedures for seamless data downloads to common statistical packages; and (4) procedures for importing data from external sources.

### Statistical analysis

Statistical analysis was performed using Stata 13 (StataCorp, College Station, TX, USA). The tests were two-sided, with a type-I error set at α = 0.05. Quantitative data were expressed as mean ± standard deviation or median [interquartile range, IQR] according to statistical distribution. The assumption of normality was studied using the Shapiro–Wilk test. Quantitative data were compared between independent groups using ANOVA or the Kruskal-Wallis test when the ANOVA assumptions were not satisfied, followed by the appropriate multiple comparison post-hoc test: Tuckey-Kramer and Dunn’s tests, respectively. The assumption of homoscedasticity was studied using the Bartlett test. The results were expressed as effect-size and 95% confidence intervals and were compared to Cohen’s recommendations (Cohen, 1988) which define effect-size bounds as small (ES: 0.2), medium (ES: 0.5) and large (ES: 0.8, “grossly perceptible and therefore large”). Comparisons between groups concerning categorical data were performed using Chi-squared or Fisher’s exact tests followed when appropriate by Marascuillo’s procedure. A logistic regression model was run in a multivariate context to determine variables associated with work-related stress (<≥70). Covariates were selected according to univariate results and clinical relevance: gender, age, professional status, tobacco and alcohol. Results were expressed as odds-ratios and 95% confidence intervals. As proposed by certain statisticians, we chose to report all the individual p-values without applying any mathematical correction for distinct tests comparing groups. Specific attention was given to the magnitude of improvement and to clinical relevance (Rothman, 1990; Feise, 2002).

## Results

### Sample description

After three months of data collection, 1,339 answers were obtained and 1,272 answers were included in the study according to inclusion and exclusion criteria. There were no missing data for VAS stress. Among the 1,272 participants, there were 821 pharmacists-in-charge (64.5%), 280 assistant pharmacists (22.0%) and 171 pharmacy technicians (13.4%). Women were more numerous than men with 66.7% (844/1,265) and the mean age was 45.1 ± 10.6. Tobacco and alcohol consumption were identified in 13.9% (174/1,250) and 72.8% (914/1,255) of participants, respectively. Regarding occupational characteristics, the median weekly working time was 45 h (IQR: 35–52). Participants received a median of 150 patients/customers per day (IQR: 100–200). These community pharmacies were located in an area with a median population of 7,000 (IQR: 2,617–25,500). The median seniority in the profession (pharmacy) was 20 years (IQR: 10–30) and 11 years (IQR: 5–20) in the community pharmacy at the time of the study. Pharmaceutical teams were composed of 1 pharmacist-in-charge (median, IQR: 1–2), 1 assistant pharmacist (median, IQR: 0.3–1.5) and 2 pharmacy technicians (median, IQR: 1.5–3.5). Most of the participants were employed under open-ended contracts (95.4%; 1,209/1,267).

### Work-related stress

The mean score of work-related stress was 52.31 ± 27.23 and 32.8% (417/1272) of individuals had a work-related stress score ≥70/100. Men were significantly more stressed than women, considering both the mean scores (54.55 ± 28.35 *vs* 51.16 ± 26.58, *p* < 0.05) and the cut-off ≥70/100 (38.5% *vs* 29.9%, *p* < 0.01). There was no difference between professional status and no relation with the age of individuals. Considering the professional environment, no difference was noted for work-related stress scores and seniority (in the present community pharmacy or seniority in the pharmacy), the number of inhabitants in the area, the number of patients or customers per day, the number of pharmacists or pharmacy technicians in the community pharmacy. However, participants with higher work-related stress scores ≥70/100 had fewer pharmacists-in-charge in the community pharmacy than lower stressed participants (scores <70/100) (1.25  ± 0.56 *vs* 1.56 ± 6.92, *p* < 0.05). Work-related stress was significantly associated with the number of weekly working hours (higher levels of stress (scores ≥70/100) *vs* lower levels of stress (scores <70/100): 48.72 ± 16.50 *vs* 43.67 ± 15.28 h per week, *p* < 0.001).

### Work-related stress and associated comorbidities

**Figure 1:**
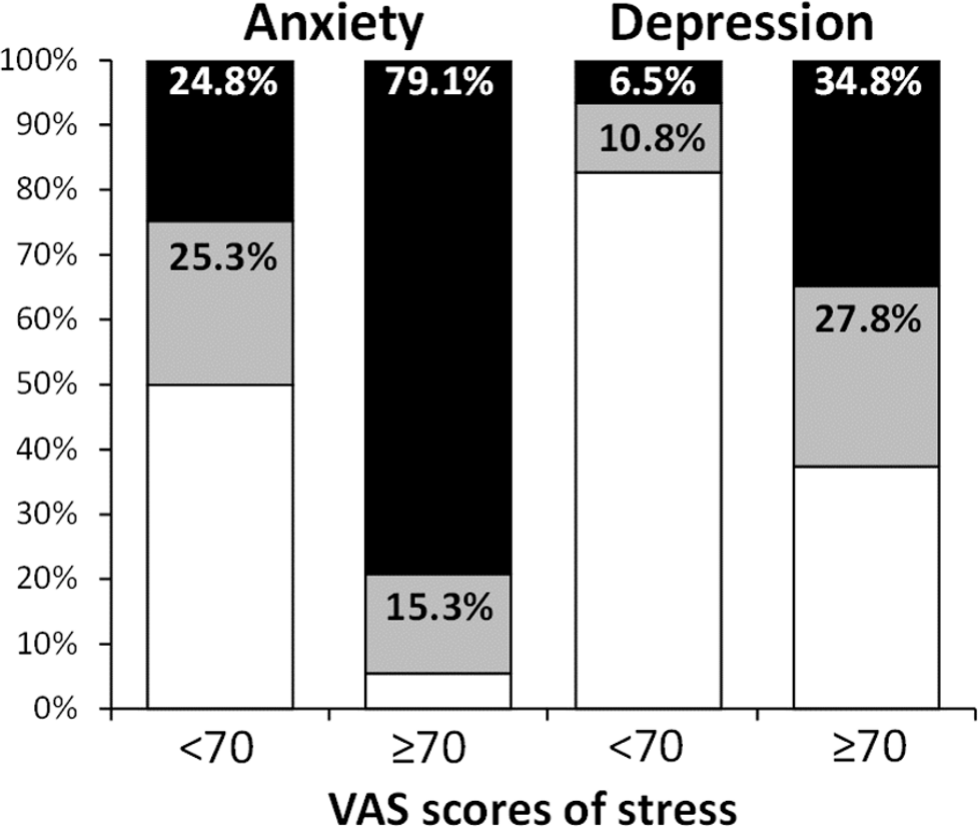
Proportion of anxiety and depression (normal, suggestive and indicative scores) according to work-related stress (<70 and ≥70/100). Anxiety and depression are presented according to normal (white), suggestive (grey) and indicative scores (black) of the HADS. Proportions of anxiety and depression scores were significantly different between higher stressed participants and lower stressed (*p* < 0.001).

Among the participants, 22.0% (280/1,272) and 42.6% (542/1,272) had suggestive or indicative scores of anxiety, respectively. Depression prevalence was lower, with 16.4% (208/1,272) and 15.8% (201/1,272) of participants presenting suggestive or indicative scores of depression, respectively. The proportions of anxiety and depression (normal, suggestive and indicative scores) were not different between pharmacists-in-charge, assistant pharmacists and pharmacy technicians (Table 1). The scores of work-related stress were significantly associated with anxiety (29.16 ± 22.93 *vs* 52.56 ± 21.55 *vs* 71.39 ± 16.0, normal *vs* suggestive *vs* indicative scores of anxiety, respectively; *p* < 0.001) and depression (42.77 ± 26.01 *vs* 68.56 ± 17.82 *vs* 76.44 ± 14.68, normal *vs* suggestive *vs* indicative scores of depression, respectively; *p* < 0.001). Likewise, proportions of indicative scores of anxiety or depression were higher for the participants with work-related stress scores ≥70/100 (Fig. 1).

**Table 1:** Participant characteristics. Quantitative results are expressed by mean ± standard deviation and qualitative results are expressed by percentage. Alcohol consumption above the WHO (World Health Organization) limits is defined as three alcoholic drinks per day for men and two alcoholic drinks per day for women. Statistical analysis was performed to compare professional status (Pharmacists-incharge vs Assistant pharmacists vs Pharmacy technicians).

	Pharmacists-in- charge *N* = 821	Assistant pharmacists *N* = 280	Pharmacy technicians *N* = 171	*p*-values
Female (*N* = 1,265)	58.1% (475)	76.9% (213)	91.2% (156)	*p* < 0.001
Age (*N* = 1,269)	48.08 ± 9.28	39.37 ± 10.83	40.17 ± 10.34	*p* < 0.001
Married/couple (*N* = 1,024)	84.0% (681)	75.8% (207)	80.5% (136)	
Single (*N* = 211)	14.1% (114)	23.8% (65)	18.9% (32)	*p* < 0.001
Widower (*N* = 18)	2.0% (16)	0.4% (1)	0.6% (1)	
Children (*N* = 1,263)	85.9% (701)	56.8% (158)	66.3% (112)	*p* < 0.001
Open ended contract (*N* = 1,267)	99.4% (816)	86.0% (240)	91.6% (153)	*p* < 0.001
Seniority—profession (*N* = 1,265)	21.77 ± 9.89	14.27 ± 10.71	19.36 ± 10.71	*p* < 0.001
Seniority—community pharmacy (*N* = 1,249)	15.10 ± 9.64	8.15 ± 7.87	12.12 ± 9.56	*p* < 0.001
Weekly working hours (*N* = 1,235)	50.84 ± 10.73	33.45 ± 7.19	33.98 ± 5.66	*p* < 0.001
Tobacco (*N* = 1,250)	13.5% (108)	10.8% (30)	21.2% (36)	*P* < 0.01
Alcohol (*N* = 1,255)	74.9% (606)	74.2% (204)	60.8% (104)	*p* < 0.001
Alcohol >WHO limits, male (*N* = 288)	9.5% (22)	6.4% (3)	10.0% (1)	NS
Alcohol >WHO limits, female (*N* = 484)	5.5% (15)	0.8% (1)	2.5% (2)	*P* < 0.05
Work-related stress scores	53.33 ± 27.14	50.84 ± 26.88	49.79 ± 28.15	NS
Work-related stress score ≥70/100	34.6% (284)	30.0% (84)	28.7% (49)	NS
Anxiety (suggestive scores)	21.2% (174)	23.6% (66)	23.4% (40)	NS
Anxiety (indicative scores)	44.7% (367)	36.4% (102)	42.7% (73)	NS
Depression (suggestive scores)	17.4% (143)	14.6% (41)	14.0% (24)	
Depression (indicative scores)	15.7% (129)	15.0% (42)	17.5% (30)	
Fatigue scores	60.65 ± 24.48	57.27 ± 24.28	57.85 ± 25.59	NS
Sleep disturbances	82.4% (673)	80.9% (225)	77.5% (131)	NS

Fatigue scores were not different between pharmacists-in-charge, assistant pharmacists and pharmacy technicians (Table 1). However, scores of work-related stress were significantly correlated to fatigue scores (*r* = 0.70, *p* < 0.001) and fatigue scores were significantly higher for participants with high levels of stress (stress scores ≥70/100) compared to participants with low levels of stress (<70/100) (77.17 ± 15.03 *vs* 50.74 ± 23.72, *p* < 0.001).

Sleep disturbances were very frequent in the participants since 81.4% (1,029/1,264) of them declared they had sleep disturbances. There was no difference between pharmacists-in-charge, assistant pharmacists and pharmacy technicians (Table 1). Sleep disturbances were strongly associated with work-related stress. The scores of work-related stress were significantly higher for participants suffering from sleep disturbances compared to participants without sleep disturbances (57.24 ± 24.97 *vs* 31.17 ± 26.62, *p* < 0.001). Likewise, among participants suffering from higher scores of work-related stress (stress scores ≥70/100), the proportion of sleep disorders was significantly higher compared to participants with low levels of stress (37.6% *vs* 12.3%, *p* < 0.001).

Participants with high levels of stress (score ≥70/100) resorted more to medical consultations to manage their work-related stress than participants with low levels of stress (17.0% *vs* 5.6%, *p* < 0.001). Similarly, participants resorting to medical consultation had higher stress scores than participants not resorting to medical consultation (71.09 ± 16.84 *vs* 50.40 ± 27.37, *p* < 0.001). Moreover, pharmacy technicians resorted more to medical consultation to manage their work-related stress than other community pharmacy professionals (7.1% *vs* 12.5% *vs* 15.3%, pharmacists-in-charge *vs* assistant pharmacists *vs* pharmacy technicians, respectively; *p* < 0.001). Participants with high levels of stress (score ≥70) took more medications to manage their work-related stress than participants with low levels of stress (42.9% *vs* 14.8%, *p* < 0.001). Participants taking medications had higher stress scores than participants not taking medications (69.07 ± 19.39 *vs* 47.19 ± 27.28, *p* < 0.001). Medication use was not different among community pharmacy professionals. Although 65.3% (192/294) of these participants taking medications were self-medicating and consequently 34.7% (102/294) had a medical prescription. The stress scores were not related to the proportion of self-medications or medical prescriptions. Pharmacists-in-charge were more self-medicated than other community pharmacy professionals (75.0% *vs* 55.6% *vs* 43.1%, pharmacists-in-charge *vs* assistant pharmacists *vs* pharmacy technicians, respectively; *p* < 0.001). Anxiolytic and hypnotic drug use was significantly associated with work-related stress scores. Anxiolytic and hypnotic drug users were more stressed (score ≥70) than non-users (anxiolytics: 65.1% *vs* 34.9%, *p* < 0.001; hypnotics: 66.7% *vs* 33.3%, *p* < 0.001) and had higher stress scores (anxiolytics: 71.97 ± 17.34 *vs* 48.75 ± 27.19, *p* < 0.001; hypnotics: 73.35 ± 19.74 *vs* 51.15 ± 27.12, *p* < 0.001). No difference in anxiolytic or hypnotic drug use was observed between pharmacists (owner and assistant) and pharmacy technicians.

The work-related stress scores were significantly higher for men with alcohol consumption over the WHO limit compared to consumption under the WHO limit (73.8 ± 18.11 *vs* 53.71 ± 28.00, *p* < 0.001). Likewise, 16.7% of men with a stress score ≥70 had an alcohol consumption over the WHO limit compared to 4.0% of men with consumptions under the WHO limit (*p* < 0.001), whereas there was no difference for women’s alcohol consumptions. A high stress score was not related to tobacco consumption. Participants with high levels of stress (score ≥70) resorted more to psychoactive drugs to manage their work-related stress than participants with low levels of stress (14.3% *vs* 5.1%, *p* < 0.001) and users of psychoactive drugs had higher stress scores than non-users (68.20 ± 20.16 *vs* 50.90 ± 27.37, *p* < 0.001).

**Figure 2:**
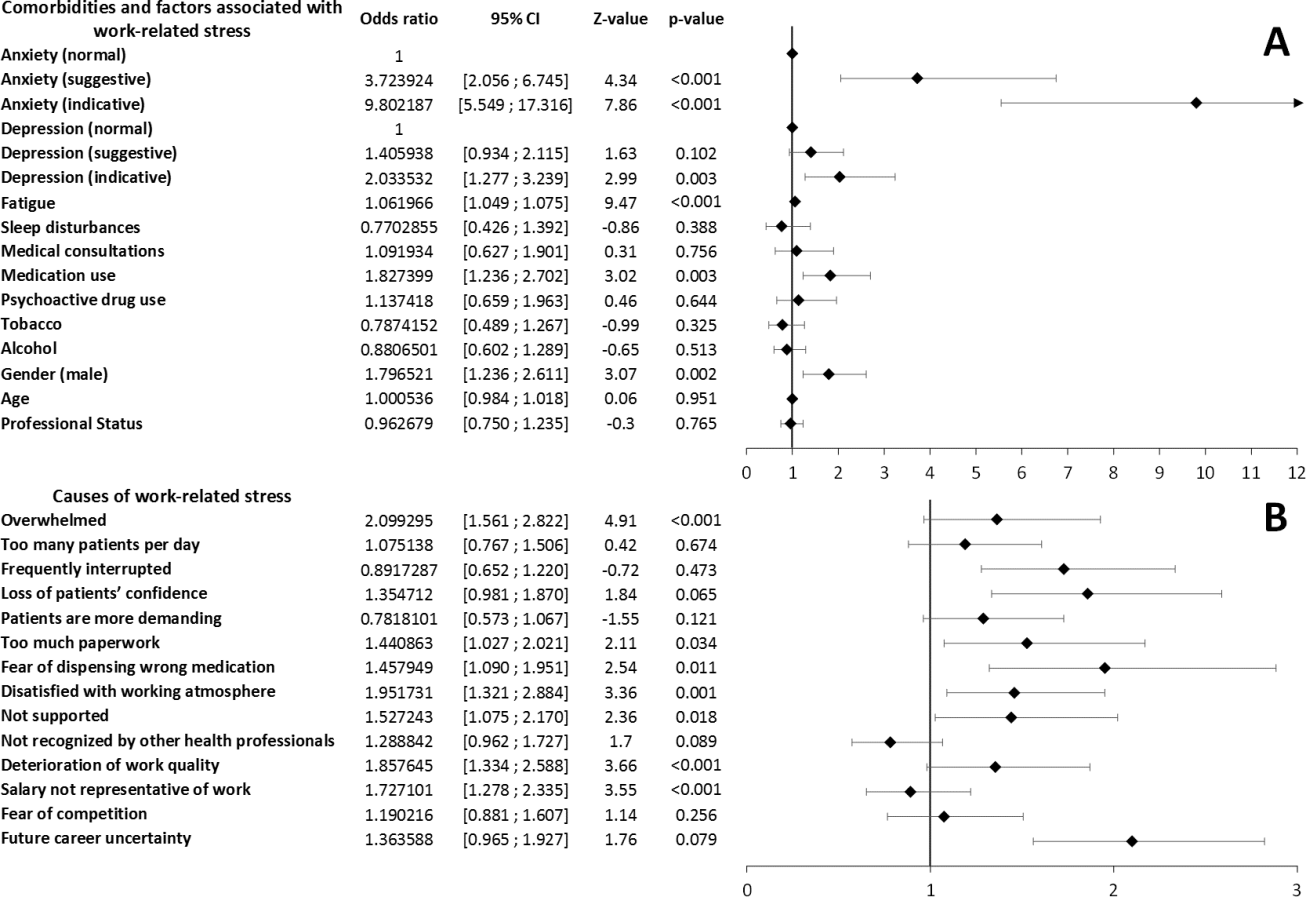
Multivariate analysis of comorbidities and factors associated with work-related stress levels (A) and causes of stress (B) associated with work-related stress levels. Multivariate analysis of causes of stress associated with work-related stress levels were adjusted to gender, age, tobacco and alcohol consumption and professional status.

The multivariate analysis of comorbidities associated with work-related stress showed significant association with anxiety (suggestive and indicative scores), depression (indicative scores), fatigue, males and medication use (Fig. 2).

### Causes of work-related stress

**Figure 3:**
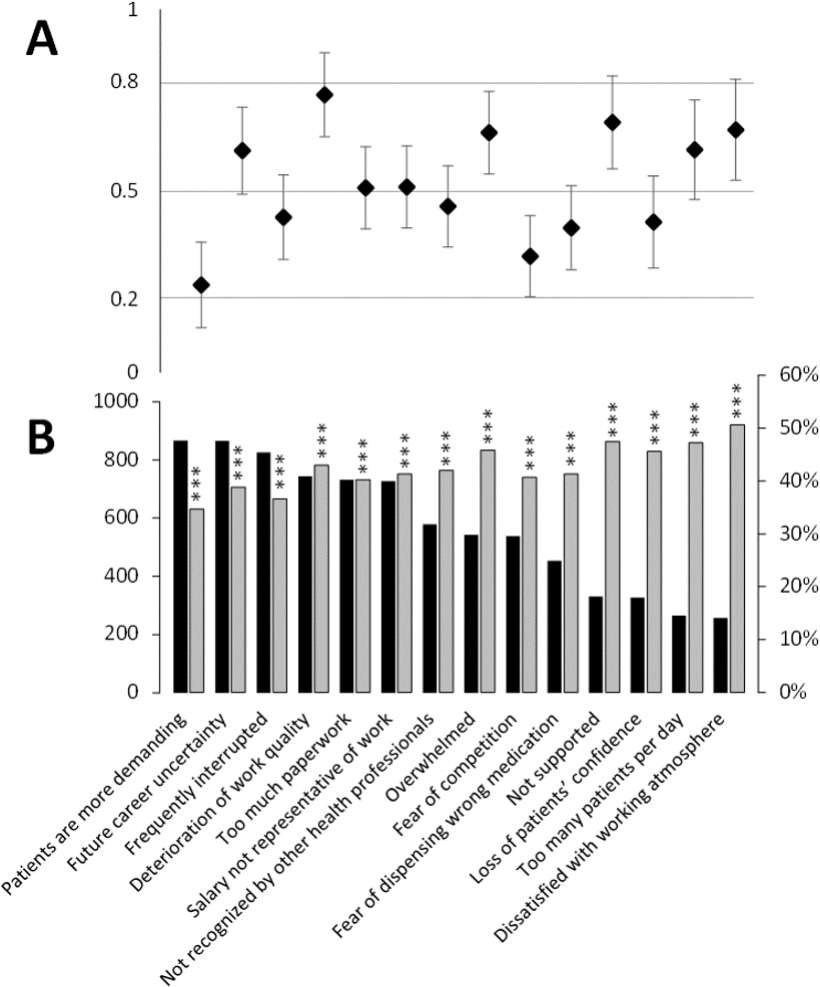
Causes of work-related stress according to stress scores ≥70/100 and effect size of stress scores for each cause of work-related stress. The occurrence of causes of work-related stress checked by the participants are presented by decreasing order from left to right (*N* = 1,272) (B, black bar). Percentages of participants who have stress scores ≥70/100 are presented for each cause of work-related stress (B, grey bar). ^∗∗∗^: *p* < 0.001, stress scores ≥70/100 *vs* <70/100 The effect size (A) defines the difference between the mean stress scores for the causes of stress *vs* unchecked causes of stress checked divided by the standard deviation. Effect size allows identifying the level of difference. Effect size <0.2 signifies no effect, 0.2–0.5 a small effect, 0.5–0.8 a medium effect and >0.8 a large effect (Cohen, 1988).

Causes of work-related stress are presented in Fig. 3. Each cause was significantly related to work-related stress scores. Quantitatively, among all these causes, more than half the participants (*N* > 700) noted the following six: “Patients are more demanding”, “Future career uncertainty”, “Frequently interrupted”, “Deterioration of work quality”, “Too much paperwork” and “Salary not representative of work”. More precisely, the effect sizes of the stress scores for each cause of work-related stress allowed identifying the biggest difference between participants with the higher scores of stress and the lower scores for each cause of stress (Fig. 3). A moderate effect was found for “Deterioration of work quality”, “Not supported”, “Dissatisfied with working atmosphere”, “Overwhelmed”, “Too many patients per day” and “Future career uncertainty”.

**Figure 4:**
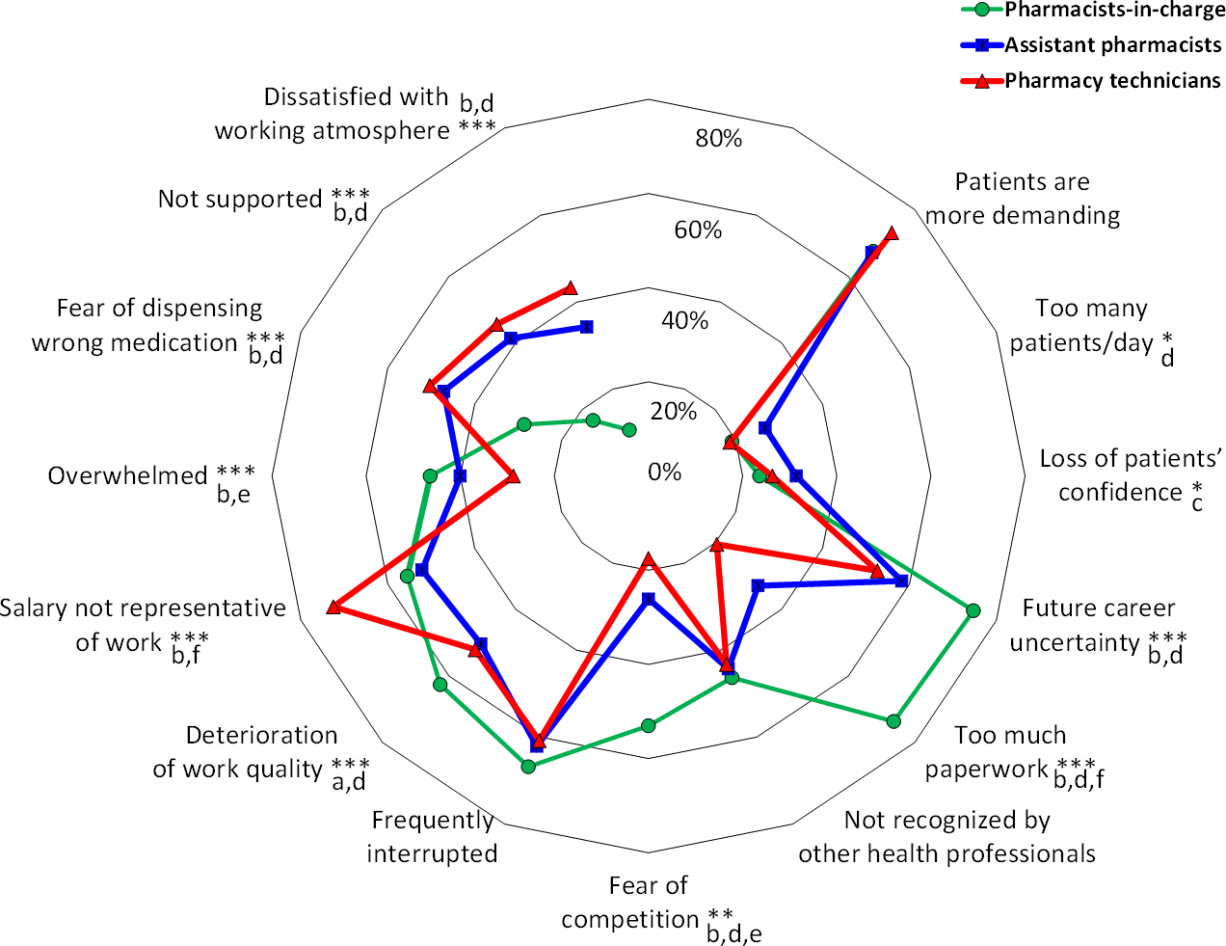
Causes of work-related stress according to professional status (pharmacists-in-charge, assistant pharmacists and pharmacy technicians). The kiviat diagram shows the percentage of participants who checked a cause of work-related stress according to their professional status (pharmacists-in-charge, assistant pharmacists and pharmacy technicians). *: *p* < 0.05, **: *p* < 0.01 and ***: *p* < 0.001 between groups; a, *p* < 0.05 (pharmacists-in-charge *vs* pharmacy technicians); b, *p* < 0.01 (pharmacists-in-charge *vs* pharmacy technicians); c, *p* < 0.05 (pharmacists-in-charge *vs* assistant pharmacists); d, *p* < 0.01 (pharmacists-in-charge *vs* assistant pharmacists); e *p* < 0.05 (assistant pharmacists *vs* pharmacy technicians); and f, *p* < 0.01 (assistant pharmacists *vs* pharmacy technicians).

However, causes of work-related stress did not seem to affect each professional in the same way (Fig. 4). Pharmacists-in-charge were typically more stressed by future career uncertainty, the burden of paperwork, fear of competition, deterioration of working conditions and work overload. Assistant pharmacists and pharmacy technicians had very similar causes of work-related stress. Assistant pharmacists were more stressed by the loss of patients’ confidence, the number of patients per day, dispensing mistakes, working atmosphere, lack of support by colleagues and future career uncertainty. Pharmacy technicians were more stressed by low salary, medication mistakes, working atmosphere, lack of support by colleagues and future career uncertainty.

Finally, multivariate analysis of stress causes associated with work-related stress indicated significant association with the feelings of being overwhelmed, too much paperwork, fear of dispensing wrong medication, working atmosphere, deterioration of work quality and salary not representative of work (Fig. 2).

## Discussion

In France, there were 27,120 pharmacists-in-charge and 27,327 assistant pharmacists in 2014 (Ordre National des Pharmaciens, 2017d). No information is available on the number of pharmacy technicians. In France, pharmacies benefit from a pharmaceutical monopoly restricting the dispensing of drugs and ownership of community pharmacies to pharmacists. French community pharmacies are all independent and are owned by one or several pharmacists-in-charge. Both pharmacists-in-charge and assistant pharmacists have a PharmD and must be registered with the National Order of Pharmacists to work. Assistant pharmacists and pharmacy technicians are employees of pharmacists-in-charge. Pharmacy technicians work under the responsibility of a pharmacist (Ordre National des Pharmaciens, 2017c).

This study identified a high level of work-related stress in a large sample of community pharmacies in France, with more than 30% of participants strongly affected. Levels of work-related stress were not different between pharmacists-in-charge, assistant pharmacists and pharmacy technicians. However in this study, men and hard workers had higher levels of stress. Nearly three quarters of pharmaceutical teams are composed of women and it is probable that the work expectations of men are higher than for women. These work expectations may be related to quantitative demand such as the number of working hours per week which affects men rather more than women, whereas women are more affected by qualitative demand such as affective load (empathy for patients) (Rivera-Torres, Araque-Padilla & Montero-Simó, 2013). Consequently, work-related stress in community pharmacies appears to be mainly related to workload which preferentially affects men.

Work-related stress was associated with several comorbidities such as anxiety, depression, fatigue and the use of medications, notably anxiolytic and hypnotic drugs. In this population with easy access to medications, it is important to underline that 65.3% of participants were self-medicating. Although there was no significant relation between stress scores and self-medication, there is reason for concern regarding the risk of medication abuse, notably with anxiolytic and hypnotic drugs. Moreover in health professionals such as physicians and medical students, self-medication is strongly embedded within the culture and related to medication abuse (Montgomery et al., 2011).

Comparing comorbidities with data from the literature is quite difficult because of the sample size and the participant profiles (age, sex). However, levels of depression seemed to be slightly higher (indicative scores of depression: 15.8%) than those found in French working populations (40–49 year old men: 10.9%, men aged ≥50 years: 14.7%, 40–49 year old women: 11.6% and women aged ≥50 years: 12.0%; *N* = 6,082 men and *N* = 5, 521 women) (Cohidon et al., 2010). Levels of anxiety were also higher in our population of pharmacists and pharmacy technicians (indicative scores of anxiety: 42.6%) compared to other French studies, 9% men and 18.4% women (*N* = 20,992) (Bocéréan & Dupret, 2014), 17% men and 25% women (*N* = 36,000) (Cohidon, 2007).

Besides its impact on health, work-related stress is associated with a considerable cost for society ranging from US$221.13 million to $187 billion (Hassard et al., 2017). This cost is primarily attributed to productivity related losses (70–90%) and to health care and medical costs (10–30%) (Hassard et al., 2017). We can also hypothesize that this work-related stress is related to a loss of productivity within community pharmacies and to a decrease of the quality of medication dispensing. It has been demonstrated that work overload with the increasing number of drug prescriptions is associated with a higher number of dispensing errors, thus compromising patient safety (Johnson et al., 2014).

The causes of work-related stress were difficult to identify among those proposed, because they were all individually related to stress scores (univariate analysis). However, a deeper statistical analysis based on effect size and multivariate analyses allowed identifying the prevalent causes of work-related stress. After these analyses, three causes were clearly identified with effect size and multivariate analyses: workload, working atmosphere and deterioration of work quality. Moreover, although the stress levels were not different between the different professional statuses, the causes of stress were clearly different between different professionals, underlining the expectations and issues for each of them. In France, community pharmacies are often small stores, where pharmacists and technicians work in close proximity with a common goal, but with different diplomas and salaries, leading to human relationship issues such as hierarchical superiority and responsibilities. Pharmacists-in-charge manifested causes of stress related to entrepreneurial burdens such as future career uncertainty, the burden of paperwork, fear of competition and work deterioration. Employees (assistant pharmacists and pharmacy technicians) expressed causes of stress related to daily pharmacy practice such as the burden of workload and the fear of dispensing mistakes, and their own perception of work, such as working atmosphere, lack of support and future career uncertainty. For example in a Canadian study, community pharmacists expressed concerns regarding having enough time for breaks or lunches, enough time to do their jobs and enough staffing support (Tsao et al., 2016).

Major changes are occurring in the world of French community pharmacies, such as the development of patient education and counseling for chronic diseases (French National Assembly and Senate, 2009), the decrease in the number of community pharmacies each year (about 200/22,000) (Ordre National des Pharmaciens, 2017b), experimentation for vaccination (flu vaccines) (Ordre National des Pharmaciens, 2017a) and the decrease in annual turnover (3–5%) (Le moniteur des Pharmacies, 2017). All these evolutions may contribute to feelings of work overload and deterioration of work quality.

Work-related stress can be prevented and managed through individual and organizational strategies (Michie, 2002). The management of work-related stress in community pharmacies has recently been reviewed (Jacobs, Johnson & Hassell, 2017). The prevention and management of work-related stress can be defined by the level of prevention (i.e., primary, secondary or tertiary), the targets (i.e., individual, individual-organization or organization) or the type of modification (i.e., socio-technical or psychosocial). At the individual level, these interventions may increase job satisfaction, well-being, autonomy and perceived stress. At the organizational level, these interventions may improve rates of absence due to sickness (Jacobs, Johnson & Hassell, 2017).

The strength of this study was the high number of participants (>1,000) which led to a good assessment of work-related stress though a simple VAS. Moreover, the high number of participants allowed identifying and discriminating comorbidities associated with stress levels. Finally, the survey was distributed by the National and Regional Orders of Pharmacists, ensuring territorial coverage of all community pharmacies.

The weaknesses of this study were, firstly, the low response rate of about 1–3% depending on professional status, which may decrease the representativeness of participants. Nonetheless, the wide distribution of the survey to community pharmacies through the National and Regional Orders of Pharmacists should limit bias. Secondly, the topic of this study, work-related stress in community pharmacies, could have over-evaluated stress levels since some of the participants could have used the survey to express their distress though this is a common reason for bias (self-selection bias) in surveys (Khazaal et al., 2014).

## Conclusion

Caution is necessary when interpreting the results of this study because of the low response rate. A third of community pharmacy teams (pharmacists-in-charge, assistant pharmacists and pharmacy technicians) suffered from work-related stress. Men were clearly more exposed to work-related stress. Work-related stress was associated with several comorbidities and health expenditures. Finally, pharmacists-in-charge, assistant pharmacists and pharmacy technicians had different causes of work-related stress associated with their status and functions.

Work-related stress should be regularly explored in each French community pharmacy and we recommend developing individual and organizational stress management in them. Since academic pharmacy degrees are mainly based on the pharmaceutical sciences (pharmacology, clinical pharmacy, etc.), it is necessary to develop additional training for stress management. Such training should be carried out by taking a global approach to both pharmacy students during pharmacy studies and to practicing pharmacists. Community pharmacies are part of the healthcare system of each country and must be supported during this period of change.

##  Supplemental Information

10.7717/peerj.3973/supp-1 Data S1 Raw data

## Additional Information and Declarations

### Data Availability

Raw data is available in the Supplemental Information.
